# Discordance for placental mesenchymal dysplasia in a monochorionic diamniotic twin pregnancy: A case report

**DOI:** 10.1002/ccr3.1662

**Published:** 2018-06-22

**Authors:** Willem Gheysen, David Strybol, Philippe Moerman, An Steylemans, Anniek Corveleyn, Luc De Catte, Isabel Couck, Liesbeth Lewi

**Affiliations:** ^1^ Department of Development and Regeneration KU Leuven University Hospitals Leuven Leuven Belgium; ^2^ Department of Obstetrics and Gynecology University Hospitals Leuven Leuven Belgium; ^3^ Department of Imaging and Pathology KU Leuven University Hospitals Leuven Leuven Belgium; ^4^ Department of Pathology University Hospitals Leuven Leuven Belgium; ^5^ Department of Obstetrics and Gynecology Ziekenhuis Netwerk Antwerpen– Campus Middelheim Antwerpen Belgium; ^6^ Department of Human Genetics KU Leuven University Hospitals Leuven Leuven Belgium; ^7^ Centrum voor Menselijke Erfelijkheid University Hospitals Leuven Leuven Belgium

**Keywords:** monochorionic twin pregnancy, placental mesenchymal dysplasia, prenatal ultrasound

## Abstract

Placental mesenchymal dysplasia (PMD) occurs in about 1 in 5000 pregnancies. The differential diagnosis between PMD and partial mole is difficult on ultrasound scan, and karyotyping plays a key role in distinguishing PMD from partial mole. Our report is the first to report on the discordancy for PMD in a monochorionic setting.

## 1 CASE REPORT

**Figure 1:**
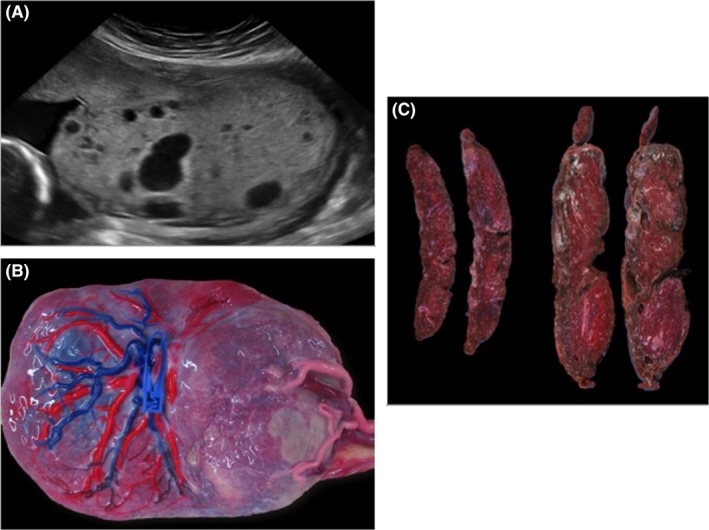
A, 2‐D ultrasound image demonstrating normal and molar aspect of the placenta of twin 1 (left) and twin 2 (right), respectively. B, Placenta after color dye injection showing the absence of vascular anastomoses. Twin 1 has an eccentric cord insertion (1 clamp‐left), whereas twin 2 has a marginal cord insertion (right). C, Macroscopic images demonstrating the normal placental tissue of twin 1 (left) and the cystic enlarged part belonging to twin (right)

A 34‐year‐old gravida 3 para 2 was referred at 20 weeks because of growth restriction in one twin of a spontaneous monochorionic diamniotic pregnancy. Twin 1 had normal amniotic fluid and appeared structurally normal (estimated fetal weight [EFW] 335 g). Umbilical artery and ductus venosus Doppler studies were unremarkable, whereas the middle cerebral artery showed decreased peak systolic velocities (19 cm/s‐0.74 multiples of the median [MoM]) suggestive of polycythemia. Twin 2 had severe growth restriction (65% growth discordance compared to twin 1, EFW 118 g) and had a pericardial effusion. Additionally, there was an anhydramnios and its bladder was empty. Doppler studies of the umbilical artery showed an absent end diastolic flow, accompanied by a reversed a‐wave of the ductus venosus and increased peak systolic velocities of the middle cerebral artery (52 cm/s, 2 MoM) suggestive of anemia. Its placental part was thickened (7 cm) and contained multiple cysts (Figure 1A). The working diagnosis was that of severe early‐onset discordant growth probably related to a chromosomal discordancy with diandric triploidy or partial mole in one of the twins with associated twin anemia polycythemia sequence. The parents were offered genetic testing by amniocentesis of twin 1 and by chorionic villous sampling of twin 2. We discussed the likely poor outcome with expectant management, the option of selective reduction of twin 2, if twin 1 was confirmed to be normal as well as a termination of the entire pregnancy. However, the parents wished to continue the twin pregnancy and therefore declined invasive testing.

Subsequent scans at 26 and 30 weeks showed an improvement of the condition of twin 2 with positive flow in the umbilical artery, a positive a‐wave in the ductus venosus, increased amniotic fluid, and a normalization of the middle cerebral artery peak systolic velocity. From 30 weeks onwards, the patient was seen twice weekly for fetal monitoring and a course of lung maturation was given.

At 34 weeks, an elective cesarean section was performed with the birth of 2 boys. Twin 1 had a birthweight of 2130 g and Apgar scores of 8, 9, 9 after 1, 5, and 10 minutes, respectively. Twin 2 weighed 970 g with Apgar scores of 7, 8, 9, after 1, 5, and 10 minutes, respectively. Both had normal hemoglobin levels. Twin 1 was hospitalized for 22 days without any major neonatal problems. Twin 2 stayed in the neonatal intensive care unit for 58 days and required nasal CPAP for 4 days and 2 units of packed cells for anemia on day 30 and day 50 after birth. Additional postnatal examinations in twin 2, such as serial brain scans, abdominal ultrasound scan, echocardiography, and metabolic tests, were normal. Conventional karyotype and single‐nucleotide polymorphism array analysis of both twins showed 46, XY and absence of pathologic copy number variants, respectively. DNA fingerprinting confirmed a monozygotic twin pregnancy. Developmental assessment by Bayley scores at the age of 5 months showed a mild developmental delay in both children.

Placental injection studies did not show any vascular anastomoses between the vascular territories (Figure 1B). Macroscopic examination showed a normal part of twin 1 and a cystic enlarged part of twin 2 (Figure 1C). Pathological examination confirmed a monochorionic diamniotic twin placenta with normal findings in the part of twin 1. In the part of twin 2, the chorionic vessels were strongly dilated. In the stem villi, the stroma was severely hydropic with the formation of central cisterns. Starting from the stem villi and extending into the intermediary and terminal villi, there was also a diffuse capillary proliferation (chorangiomatosis). No trophoblastic hyperplasia was seen. In the stem villi, the trophoblastic cells were p57 positive, whereas the stromal fibroblasts were p57 negative. Pathology therefore confirmed discordancy for placental mesenchymal dysplasia in a monochorionic placenta.

## 2 DISCUSSION

Placental mesenchymal dysplasia (PMD) occurs in about 1 in 5000 pregnancies and is 4 times more common in girls than in boys. PMD and partial moles look similar on ultrasound scan. The differential diagnosis is important because partial mole is a lethal fetal abnormality and carries a small risk to convert into malignant trophoblastic disease, whereas PMD may still result in a normal live birth and is not associated with malignancy. However, PMD increases the risks of adverse pregnancy outcome and is associated with preterm birth (52%), growth restriction (33%), genetic syndromes such as the Beckwith‐Wiedemann syndrome (28%) and fetal death (13%).^1^

Karyotyping plays a key role in distinguishing PMD from partial mole. In our case, we therefore offered genetic testing. In the setting of a monochorionic diamniotic twin pair with one abnormal twin, both sacs should be sampled to exclude rare discordances in chromosomal anomalies, a phenomenon known as heterokaryotypic monozygotic twinning.^2^ However, in our case, the anhydramnios in one sac precluded a double amniocentesis and we therefore offered amniocentesis for the normal twin together with a chorionic villous sampling of the molar‐like placental part of the growth‐restricted twin.

Partial moles of paternal origin usually result from the fertilization of a single egg by 2 spermatozoa leading to diandric triploidy (69 chromosomes, 1 set of maternal and 2 sets of paternal origin). In contrast, in PMD, the fetal karyotype is usually normal (biparental diploid, 46 chromosomes, 1 set from each parent), whereas the placenta typically is mosaic with chorionic mesoderm, membranes and vessels being diandric diploid (46 chromosomes, all from paternal origin) and with the trophoblastic cells being normal biparental diploid.^3^ In our case, the parents declined invasive testing, but the absence of any structural anomalies and the evolution into the third trimester made the diagnosis of partial mole less plausible. Typical for PMD, the trophoblastic cells were normal biparental diploid and thus P57 positive on immunohistochemistry, whereas the stromal cells were diandric and thus p57 negative. This is because only the maternal p57 gene is expressed, whereas the paternal p57 gene is imprinted and thus transcriptionally silenced.

As these male twins were confirmed to be monozygotic, one possible mechanism to explain diandric/biparental mosaicism in part of the placenta may be that the embryo itself resulted from a double fertilization by one X‐ and one Y‐bearing spermatozoon resulting in a diandric triploidy, 96, XXY. Subsequently, this triploid conception may have divided into a normal biparental cell line (46, XY) and a diandric one (46, XX) that remained confined to the placental part of the smaller twin.^3^ Alternatively, one normal egg may have been fertilized by an Y‐bearing spermatozoon resulting in a normal 46, XY cell line, whereas simultaneously an empty egg was fertilized by an X‐bearing sperm resulting in a diandric 46, XX cell line. Subsequently, this abnormal cell line may have fused with the placental part of the smaller twin to form a chimeric diandric/biparental placenta.^4^

Two previous case reports described PMD in twin pregnancies. One reported on PMD in one of a dichorionic pair, resulting in an elective birth at 35 weeks for growth restriction of the affected twin, who had a normal development at 18 months of age.^4^ The other case was a monochorionic diamniotic twin pair with PMD affecting the entire placenta. Despite mild growth discordance, the pregnancy progressed uneventfully until 25 weeks, when an unexpected demise of both twins was diagnosed.^5^ Our case is the first to report discordancy for PMD in a monochorionic setting.

As mentioned above, PMD is associated with increased risks of fetal death. In a monochorionic pair, death of one twin may lead to demise or brain damage of the cotwin because both are connected through vascular anastomoses and the surviving twin may exsanguinate into the body and placenta of its demised twin.^6^ In this particular case, we estimated the chances of subsequent demise of the growth‐restricted twin to be extremely high because of the 68% growth difference, the abnormal Doppler findings with anhydramnios, and the suspicion of twin anemia polycythemia sequence. We therefore offered a reduction by bipolar coagulation of cord of the growth‐restricted twin to protect the normal growing twin.^7^ Nevertheless, the parents chose to continue with the twin pregnancy.

Although the growth difference remained, Doppler findings and amniotic fluid levels improved in the smaller twin, who continued to grow along its centile. We suspect that twin anemia polycythemia syndrome resolved spontaneously and tiny vascular anastomoses present at 20 weeks disappeared with progressive placental growth, which explains the absence of any anastomoses at the time of birth. Because of the risk of unpredictable fetal death with possible consequences for the appropriately grown cotwin, an elective section was performed at 34 weeks.

To conclude, our case shows that discordancy for PMD may occur in the setting of a monochorionic twin pregnancy. The shared circulation complicates management, because demise of the twin with the abnormal placenta may result in death or handicap of its cotwin.

## CONFLICT OF INTEREST

None declared.

## AUTHORSHIP

All Authors listed on the title page have contributed to the work, have read the manuscript and approved to its publication.
